# Medical Department of the University of Buffalo

**Published:** 1854-11

**Authors:** 


### EDITORIAL DEPARTMENT.

*Medical Department of the University of Buffalo.*—The dissecting term of this institution has just opened with a prospect of an increase in the number of the class over that of last year. The improvement is not in numbers only, but in the character of the students. Judging from a casual glance at those already assembled, the change for the better which has for some years past been making in the appearance of medical classes is still progressing.

It would be interesting to study the causes of the present condition of medical schools. They have fewer students than eight or ten years ago, while at the same time more men of means, education, and gentlemanly affinities, are seeking the medical profession. It is probable that the cheap and gratuitous schools absorb a large portion of that class of students who crowded the lecture-rooms of all colleges a few years ago, but it is equally demonstrable that uneducated boors are not as common anywhere. Those choosing medicine merely as a means of livelihood, have been drawn off by commercial enterprize, by the new avenues of employment opened by our increasing railroad business, by Californian emigration, and by the general prosperity of all trades and avocations. The regulations of the National Association requiring a preliminary classical education have also had an influence.

It is a pity that while all these causes are working together for good to the profession at large, the colleges should be the only sufferers. But it is evident that too many colleges are already in existence, that the laws of supply and demand are applicable to medical education, and that the college edifices which have been erected in times of "expansion," to use a commercial term, must some of them remain unoccupied, or be converted to other purposes. It is equally evident that those schools must fall first which are destitute of means of clinical instruction.

An instance of this is found in the fate of the Willoughby school. Uncommon energy and perseverance had raised this institution to a very respectable standing, and given to it large classes. Its original faculty, however, rebelled at the inconveniences and deficiencies of a small country village, and throwing up their appointments they removed in a body to Cleveland, where they established the Cleveland Medical College under the charter of the Western Reserve College. This occurred in 1843. Since that time few schools have been more prosperous than it, thus confirming the policy which dictated the removal.

The trustees at Willoughby reorganized with a new and very able faculty. The general rush into the profession which took place at that time, enabled the school to go on for four sessions with large classes and every outside evidence of a high state of prosperity. The new men were, however, too shrewd to stay long enough to be defeated, and, breaking up the school at the end of a session of 130 students, they followed the example of their predecessors, and removed to Columbus, establishing there the Starling Medical College.

This new enterprize also prospered, and the Willoughby school was converted into a female seminary, which still flourishes.

Such must, eventually, be the fate of all the country schools. They do not die for lack of talent or industry, but the tendency of the profession is to centralization in the large cities. We should regret to see any one city in the United States assume the monopoly of medical teaching and influence. Our country is too large, and its interests and sympathies too diverse, to permit this, and we may close our discursive article with the proposition with which we started, that the laws of supply and demand must govern the issue of all schemes in medical teaching. H.

